# Hackflex: low-cost, high-throughput, Illumina Nextera Flex library construction

**DOI:** 10.1099/mgen.0.000744

**Published:** 2022-01-11

**Authors:** Daniela Gaio, Kay Anantanawat, Joyce To, Michael Liu, Leigh Monahan, Aaron E. Darling

**Affiliations:** ^1^​ iThree Institute, University of Technology Sydney, Sydney, NSW, Australia

**Keywords:** high-throughput sequencing, Illumina sequencing, library preparation, metagenomics, multiplex, Nextera Flex

## Abstract

We developed a low-cost method for the production of Illumina-compatible sequencing libraries that allows up to 14 times more libraries for high-throughput Illumina sequencing to be generated for the same cost. We call this new method Hackflex. The quality of library preparation was tested by constructing libraries from *Escherichia coli* MG1655 genomic DNA using either Hackflex, standard Nextera Flex (recently renamed as Illumina DNA Prep) or a variation of standard Nextera Flex in which the bead-linked transposase is diluted prior to use. In order to test the library quality for genomes with a higher and a lower G+C content, library construction methods were also tested on *Pseudomonas aeruginosa* PAO1 and *Staphylococcus aureus* ATCC 25923*,* respectively. We demonstrated that Hackflex can produce high-quality libraries and yields a highly uniform coverage, equivalent to the standard Nextera Flex kit. We show that strongly size-selected libraries produce sufficient yield and complexity to support *de novo* microbial genome assembly, and that assemblies of the large-insert libraries can be much more contiguous than standard libraries without strong size selection. We introduce a new set of sample barcodes that are distinct from standard Illumina barcodes, enabling Hackflex samples to be multiplexed with samples barcoded using standard Illumina kits. Using Hackflex, we were able to achieve a per-sample reagent cost for library prep of A$7.22 (Australian dollars) (US $5.60; UK £3.87, £1=A$1.87), which is 9.87 times lower than the standard Nextera Flex protocol at advertised retail price. An additional simple modification and further simplification of the protocol by omitting the wash step enables a further price reduction to reach an overall 14-fold cost saving. This method will allow researchers to construct more libraries within a given budget, thereby yielding more data and facilitating research programmes where sequencing large numbers of libraries is beneficial.

## Data Summary

Scripts used for the analysis can be found at https://github.com/GaioTransposon/Hackflex. Sequencing data has been deposited with the National Center for Biotechnology Information (NCBI) under BioProject accession number PRJNA549801. Supplementary material can be found on Figshare: https://figshare.com/s/0f1a9268d57948594f7e.

**Impact Statement**

Genomic epidemiology, powered by high-throughput genome sequencing of large numbers of samples, has proven to be an incredibly powerful approach to understand pathogens, antimicrobial resistance, and their spread through the environment and human populations. High-throughput sequencing technologies, however, have been largely driven by the human genomics market, where the per-sample cost of sequencing library preparation is relatively small. For microbial studies, the per-sample cost of library preparation using commercial kits can be so high that it heavily dominates the overall cost of genomic epidemiology, limiting studies to smaller sample sizes than would otherwise be possible. To address this, we introduce Hackflex, a method to produce sequencing libraries in a cost-effective manner at high sample counts. We provide a protocol and characterize the performance of the method, describing its strengths and limitations, and the limitations of our characterization. The library preparation method is readily applicable to both isolate genome and moderate-complexity metagenome sequencing, such as mammalian gut metagenomes. When applied at full scale, Hackflex can yield a 14-fold reduction in per-sample library preparation costs, enabling thousands of microbial genomes to be sequenced in a single run of current generation Illumina NovaSeq instruments.

## Introduction

The original Nextera protocol provided an easy to use and flexible means for generating Illumina-compatible shotgun libraries. When applied at scale, however, the Nextera reagents at list price could become prohibitively expensive for projects with large sample counts and low sequencing requirements per sample. Previous work demonstrated that it was possible that by reducing the reaction volume, the per-sample library cost could be greatly reduced [1, 2], thereby facilitating the processing of large sample batches. In 2017, Illumina introduced a new type of Nextera kit, called Nextera Flex (recently renamed as Illumina DNA Prep), and subsequently discontinued the original Nextera kits for which cost-reducing strategies had been developed. The Nextera Flex kits use bead-linked transposases (BLTs) to fragment and tag DNA with adapter sequences. The tagmentation technique allows the incorporation of defined adapter sequences, enabling barcoded primers to anneal and be extended through tagmented DNA fragments in subsequent PCR amplification and sequencing reactions [3]. The new Nextera Flex kits have been shown to yield greatly improved data quality relative to the original Nextera and Nextera XT kits [4]. However, the existing protocols based on reduced reaction volumes [2, 3, 5] cannot be directly applied with the new Nextera Flex kit; hence, we developed an adaptation of those protocols that allows them to work with the Nextera Flex kit protocol, without negative impacts to performance for typical applications such as *de novo* microbial genome assembly.

**Fig. 1.**
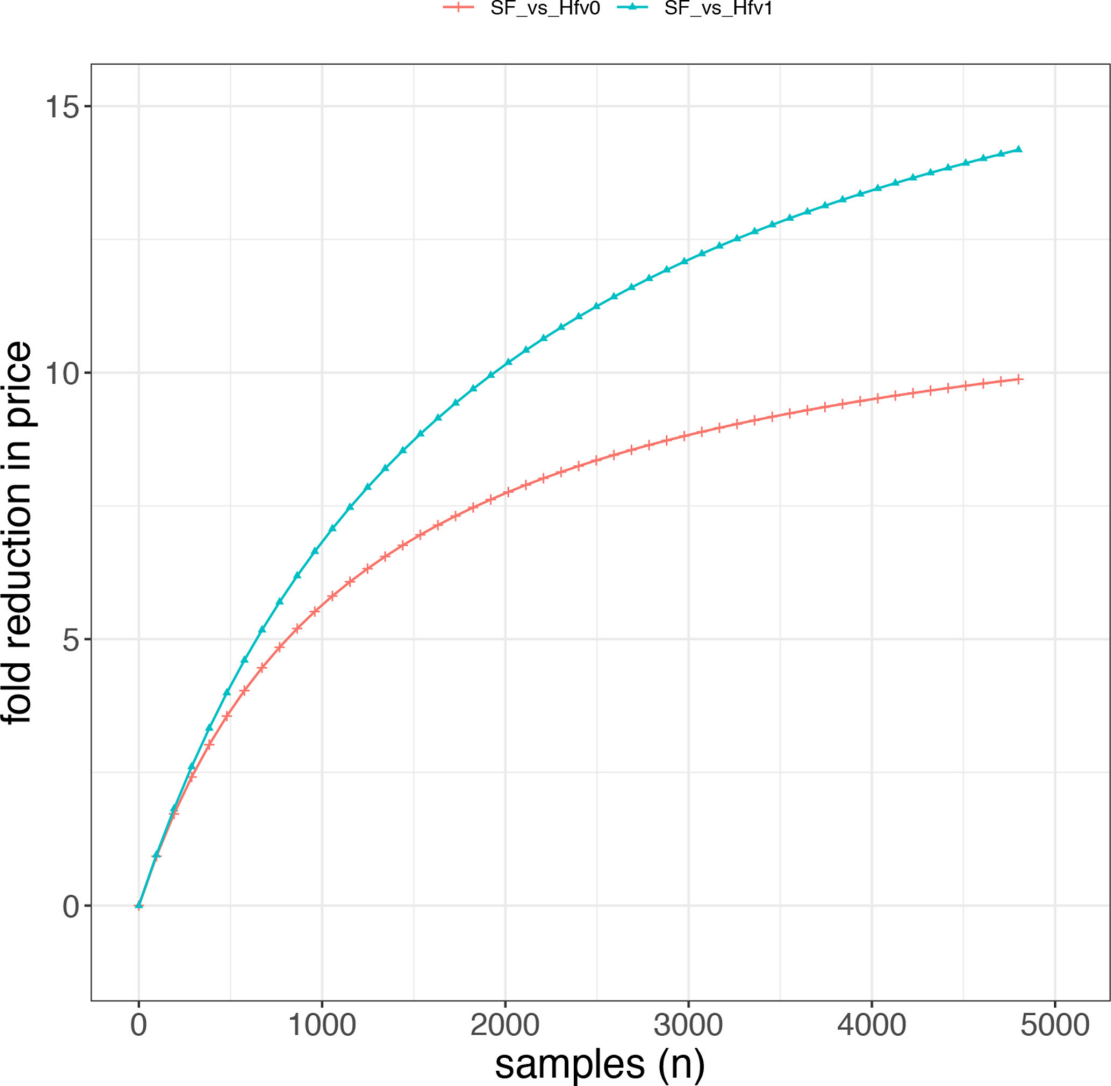
Price fold difference. Fold difference in price between standard Flex and Hackflex v0 and between standard Flex and Hackflex v1. Differently from Hackflex v0, Hackflex v1 uses half the amount of the polymerase (1 µl instead of 2 µl) and the TWB wash step is omitted.

In this work, we introduce a low-cost variant of the Nextera Flex protocol that we call Hackflex. In addition to diluting the BLTs, we propose a simplified protocol that replaces all other reagents with components readily available from third-party sources, leading to a 9.87-fold and a 14.17-fold price reduction, with an earlier version of Hackflex (v0) and with the current version of Hackflex (v1), respectively (Fig. 1, Table S1, available with the online version of this article). We present our Hackflex protocol and compare the quality of the resulting data to the standard Nextera Flex kit protocol and to a 1 : 50 bead diluted version of the standard Nextera Flex protocol. We compare the protocols in terms of read quality, uniformity of coverage, coverage of low coverage regions, G+C coverage bias and insert size length. We additionally design 96 barcode sequences that allow the generation of 9120 combinations and we test their performance. Finally, we prepare Hackflex libraries with longer fragment sizes, and demonstrate that large insert Hackflex libraries can lead to improved genome assembly for some microbes.

## Methods

### Genomic DNA preparation

Genomic DNA of three different bacteria were used in this study: *Escherichia coli* strain MG1655, *Pseudomonas aeruginosa* strain PAO1 and *Staphylococcus aureus* strain ATCC 25923. For the *E. coli* MG1655 strain, the reference genome used in this study differs from the original *E. coli* MG1655 strain sequenced by Blattner *et al*. [6], most notably as it contains a pBAD plasmid. Independent reference assemblies of the *E. coli* MG1655 stain, *P. aeruginosa* strain PAO1 and *S. aureus* strain ATCC 25923 were used (see the sections ‘*Nanopore library preparation and sequencing’* and *‘Generation of reference assemblies*’). For DNA extraction, high molecular mass genomic DNA was extracted from freshly cultivated cells of this strain using the Qiagen DNeasy UltraClean microbial kit according to the manufacturer’s instructions. Briefly, 20 ml overnight culture was centrifuged at 3200 relative centrifugal force (RCF) (for 5 min to obtain a cell pellet. Pellets were washed with 5 ml sterile 0.9 % sodium chloride solution, and then resuspended in 300 µl PowerBead solution before continuing with the kit manufacturer’s protocol. The final genomic DNA was eluted with a 50 µl elution buffer pre-warmed to 42 °C. The concentration of isolated DNA samples was measured using a Qubit 2.0 (Thermo Fisher Scientific) and diluted in water.

### Library descriptions

Below, we describe the generation of libraries using different protocols and different sources of genomic DNA. The two-letter prefix of each library indicates the source of genomic DNA used (Ec for *E. coli *; Pa for *P. aeruginosa *; Sa for *S. aureus *). The next two-letter prefix denotes the protocol used (SF, standard Flex; HF, Hackflex). For standard Flex libraries, the type of dilution used is described with 1 or 1 : 50, denoting no dilution (1) or a 1 : 50 dilution (1 : 50) (e.g. ‘Ec.SF.B1’ versus ‘Ec.SF_1 : 50.B1’). Exceptions to the standard protocol are denoted by the letters that follow, up to the suffix. For example, ‘Ec.SF_PS.B2’ indicates that the standard Flex protocol is used, with PrimeSTAR GXL polymerase (Takara) as an exception to the standard Flex protocol. The suffix to the library name indicates the source of the sequencing batch (e.g. ‘Ec.SF.B2’ where B2 stands for batch number 2). Each library preparation condition is shown schematically in Table 1.

**Table 1.** List of libraries and summary of library preparation conditions

Library name	Barcode	Reagent	Polymerase	Ann. (°C)	Ext. (°C)	Species	BLT dilution	TWB	PCR cycles and input DNA (ng)	MiSeq cycles
Ec.SF.B1	v0(t)	SF	EPM	62	68	* E. coli *	1	na	5/200	600
Ec.SF_1 : 50.B1	v0(t)	SF	EPM	62	68	* E. coli *	1 : 50	na	12/10	600
Ec.SF.B2	v0	SF	EPM	62	68	* E. coli *	1	na	5/200	600
Ec.SF_1 : 50.B2	v0	SF	EPM	62	68	* E. coli *	1 : 50	na	12/10	600
Ec.SF_PS.B2	v0	SF	PS 2 µl	62	68	* E. coli *	1	na	5/200	600
Ec.HF.B3	v0	HF	PS 1 µl	62	68	* E. coli *	1 : 50	Y	12/10	600
Ec.HF_55A.B2	v0	HF	PS 2 µl	55	68	* E. coli *	1 : 50	Y	12/10	600
Ec.HF_06x.B3	v1	HF	PS 1 µl	62	68	* E. coli *	1 : 50_0.6×	N	12/10	600
Ec.HF-barcode_v0(t).B0	v0(t)	HF	PS 2 µl	62	68	* E. coli *	1 : 50	Y	12/10	300
Ec.HF-barcode_v1.B3	v1	HF	PS 1 µl	62	68	* E. coli *	1 : 50	N	12/10	600
Pa.SF.B1	v0(t)	SF	EPM	62	68	* P. aeruginosa *	1	na	5/200	600
Pa.SF_1 : 50.B1	v0(t)	SF	EPM	62	68	* P. aeruginosa *	1 : 50	na	12/10	600
Pa.HF.B2	v0(t)	HF	PS 2 µl	62	68	* P. aeruginosa *	1 : 50	Y	12/10	600
Pa.HF_55A.B2	v0	HF	PS 2 µl	55	68	* P. aeruginosa *	1 : 50	Y	12/10	600
Pa.HF_55A72E.B2	v0	HF	PS 2 µl	55	72	* P. aeruginosa *	1 : 50	Y	12/10	600
Pa.HF_06x.B3	v1	HF	PS 1 µl	62	68	* P. aeruginosa *	1 : 50_0.6×	N	12/10	600
Sa.SF.B1	v0(t)	SF	EPM	62	68	* S. aureus *	1	na	5/200	600
Sa.SF_1 : 50.B1	v0(t)	SF	EPM	62	68	* S. aureus *	1 : 50	na	12/10	600
Sa.HF.B2	v0	HF	PS 2 µl	62	68	* S. aureus *	1 : 50	Y	12/10	600
Sa.HF_55A.B2	v0	HF	PS 2 µl	55	68	* S. aureus *	1 : 50	Y	12/10	600
Sa.HF_06x.B3	v1	HF	PS 1 µl	62	68	* S. aureus *	1 : 50_0.6×	N	12/10	600

N, No; na, Not applicable; PS, PrimeSTAR HS DNA Polymerase (Takara); Y, Yes.

### Nextera Flex library preparation

We first created a set of libraries using the standard protocol of Nextera Flex (referred to as standard Flex, abbreviated as SF). Each standard Flex library was constructed using all standard kit reagents from the Nextera DNA Flex library prep kit (Illumina), following the manufacturer’s protocol. Briefly, 200 ng input DNA in 30 µl nuclease-free water was tagmented by adding 10 µl BLT and 10 µl TB1 solution. Each sample was then incubated in the thermocycler at 55 °C for 15 min, then held at 10 °C. After the incubation, 10 µl TSB solution was added into the tagmentation reaction, and the sample was incubated at 37 °C for 15 min, then held at 10 °C. Each sample was then transferred to the magnetic stand to isolate the DNA–BLT complex. The DNA–BLT complex was washed with 100 µl Tagment Wash Buffer (TWB) solution three times. The PCR reaction for library amplification was prepared by mixing 20 µl enhanced PCR mix (EPM) with 20 µl nuclease-free water. The mixture was added into the DNA–BLT complex. Five microlitres of each i5 and i7 adapter was added into the PCR reaction. The final volume of the PCR reaction was 50 µl. PCR conditions were set to 68 °C for 3 min, 98 °C for 3 min; followed by five cycles of 98 °C for 30 s, 62 °C for 30 s, 68 °C for 2 min; 68 °C for 1 min; and held at 10 °C. After library amplification, the sample tube was placed onto the magnetic stand. Forty-five microlitres of the PCR supernatant was mixed with 85 µl diluted PB reagent (45 µl PB solution diluted in 40 µl Resuspension Buffer (RSB) solution; the final ratio of beads to the PCR supernatant is 0.53×), then incubated at room temperature for 5 min. The sample tube was then placed on the magnetic stand, and 125 µl supernatant was transferred into a new sample tube containing 15 µl undiluted PB (the ratio of the beads to the starting PCR supernatant is 0.7×). The sample was mixed and incubated at room temperature for 5 min, then the tube was placed on the magnet. The supernatant was discarded, and the beads were washed with 200 µl fresh 80 % ethanol twice. The beads were left to air-dry at room temperature and were resuspended in 32 µl RSB solution. The beads were incubated at room temperature for 2 min. The sample tube was placed on the magnet, and finally 30 µl eluted library was transferred into a new sample tube. The concentration of eluted library and the library size were measured using a Qubit high-sensitivity (HS) dsDNA kit (Thermo Fisher Scientific) and a high sensitivity Bioanalyzer chip (Agilent Technology), respectively. In total, there were four standard Flex libraries: two from *E. coli* genomic DNA (referred to as: ‘Ec.SF.B1’ and ‘Ec.SF.B2’), one from *P. aeruginosa* genomic DNA (‘Pa.SF.B2’) and one from *S. aureus* genomic DNA (‘Pa.SF.B2’).

The first step in Hackflex development was to test whether the BLT beads can be diluted. Diluted standard Flex libraries were generated by following the standard Nextera Flex protocol using the standard reagents (as described above). The quantity of all reagents used in the reaction were unchanged, except for the BLT beads which were diluted 1 : 50 with nuclease-free water prior to use (referred to as 1 : 50 diluted Flex abbreviated as ‘SF_1 : 50’). The amount of input genomic DNA was adjusted from 200 to 10 ng in proportion to the amount of BLT in the reaction, and the number of PCR cycles was increased from 5 to 12 to reflect the amount of DNA–BLT complex in the library amplification step. Of these diluted libraries, we generated a total of four libraries: two libraries from *E. coli* genomic DNA (‘Ec.SF_1 : 50.B1’ and ‘Ec.SF_1 : 50.B2’), one library from *P. aeruginosa* genomic DNA (‘Pa.SF_1 : 50.B2’) and one library from *S. aureus* genomic DNA (‘Sa.SF_1 : 50.B2’).

As the polymerase used to generate Hackflex libraries is PrimeSTAR GXL polymerase (Takara), we assessed the impact of the use of a different polymerase in standard Flex libraries, by generating one standard Flex library from *E. coli* genomic DNA, where EPM (Illumina) was replaced with PrimeSTAR GXL polymerase (Takara). We refer to this library as ‘Ec.SF_PS.B2’.

All standard Flex and modified standard Flex libraries described above were purified, pooled in equal molarity, diluted to 4 nM and quality-control checked on the Bioanalyzer (Agilent Technologies). The final pool was sequenced on Illumina MiSeq platform 2×300 bp using the MiSeq reagent kit v3 (600 cycles PE) cartridge (Illumina).

### Hackflex library preparation

Hackflex libraries were prepared using laboratory-made and adapted reagents from the Nextera DNA Flex library prep kit (Illumina) (Table S1). All incubation temperatures and times used in the Hackflex protocol were the same as in the standard Flex protocol except for the PCR amplification step, which is optimized for the polymerase.

Briefly, in the tagmentation reaction, BLT beads were diluted 1 : 50 with nuclease-free water (Invitrogen). Ten nanograms of input genomic DNA in 10 µl ultrapure water (Invitrogen) was mixed with 10 µl 1 : 50 diluted BLT and 25 µl 2× laboratory-made tagmentation buffer [20 mM Tris (pH 7.6) (Chem-Supply, Australia), 20 mM MgCl_2_ (Sigma-Aldrich) and 20 % (v/v) dimethylformamide (DMF) (Sigma-Aldrich)]. The final volume of the tagmentation reactions was 45 µl. The sample was incubated at 55 °C for 15 min, then held at 10 °C. To stop the tagmentation, 10 µl 0.2 % SDS (Sigma-Aldrich) was added into the sample, instead of using TSB. The sample was then incubated at 37 °C for 15 min and then held at 10 °C.

Once the tagmentation was completed, the beads were washed three times using 100 µl washing solution [0.22 µm MF-Millipore membrane filtered solution of 10 % PEG 8000 (Sigma-Aldrich), 0.25M NaCl (Chem-Supply, Australia) in Tris-EDTA buffer (TE) (Sigma-Aldrich)], instead of TWB. In the later adaptation of the Hackflex protocol (v1), this wash step was found to be unnecessary and, therefore, was removed.

For library amplification, EPM master mix was replaced with the PrimeSTAR GXL DNA polymerase kit (Takara), following the manufacturer’s protocol. Each PCR reaction contains 10 µl 5× GXL buffer, 4 µl 10 mM dNTPs, 2 µl PrimeSTAR GXL polymerase and 19 µl nuclease-free water. In a later adaptation of the Hackflex protocol (v1), the amount of PrimeSTAR GXL polymerase used in the reaction was reduced to 1 µl, and the amount of nuclease-free water was increased to 20 µl. The PCR mix was added into washed BLT beads. Barcodes were designed to be used for Hackflex libraries (described below). Five microlitres of each 10 µM custom-synthesized, 96-well plate Illumina adapter oligos i5 and i7 was added to a final concentration of 1 µM to each reaction. The final volume for the PCR reaction was 45 µl. Library amplification was performed with different conditions from the manufacturer’s recommended protocol, as follows: 3 min at 68 °C; 3 min at 98 °C; 12 cycles of 45 s at 98 °C, 30 s at 62 °C, 2 min at 68 °C; 1 min at 68 °C; and hold at 10 °C. After library amplification, the samples were individually cleaned up. Size selection and purification of the library followed, replacing reagents SPB and RSB with equal volumes of SPRIselect beads (Beckman Coulter) and ultrapure water (Invitrogen), respectively. The concentration of the library was measured with the Qubit HS dsDNA kit (Thermo Fisher Scientific). Fragment size distribution was assessed using the high sensitivity DNA kit on the Bioanalyzer (Agilent Technologies). Three libraries were created using the Hackflex protocol described above. These libraries were Ec.HF.B3, Pa.HF.B2 and Sa.HF.B2.

We have created two sets of 96 libraries to test the performances of barcodes v0 and v1 (described in ‘*Barcode design*’ below). The libraries are Ec-HF-barcode_v0(t).B2 and Ec-HF-barcode_v1.B3, respectively. Briefly, 96 libraries were created using Hackflex described above from *E. coli* genomic DNA. After library amplification, 3 µl each library was pooled into one tube. Then, the entire pool was cleaned up using SPRIselect beads following the protocol described above. The quality of the pooled library was assessed using the Qubit HS dsDNA kit (Invitrogen) and the Bioanalyzer (Agilent). Please refer to the ‘*Barcode design*’ section below for a description of the barcode designs. For sequencing, the final library was diluted and denatured following the manufacturer’s instructions, then 4 pM pooled library with 5 % PhiX v3 control (Illumina) was loaded onto an Illumina MiSeq instrument and sequenced using MiSeq v3 chemistry, generating 2×300 bp paired-end reads.

### Hackflex v1 protocol changes

As mentioned above, the Hackflex protocol v1 includes two differences from the Hackflex protocol v0 described above. These differences consist of: (i) the elimination of the TWB washing step, and (ii) the reduction of the amount of PrimeSTAR GXL DNA polymerase (Takara) from 2 µl to 1 µl per reaction.

### Additional Hackflex libraries

Different polymerases have their own optimized annealing and extension temperatures. In order to fine-tune the annealing and extension temperatures in the Hackflex protocol, we generated three libraries using an annealing temperature of 55 °C, instead of 62 °C (‘Ec.HF_55A.B2’, ‘Sa.HF_55A.B2’ and ‘Pa.HF_55A.B2’) and one library from *P. aeruginosa* genomic DNA, where both annealing and extension temperatures were modified (55 °C annealing and 72 °C extension instead of 68 °C; library referred to as ‘Pa.HF_55A72E.B2’).

An additional three libraries were prepared following the Hackflex protocol with the aim of producing libraries with longer fragments. For this purpose, all steps of the Hackflex protocols were followed as previously described, except for the library purification clean-up step, where the size selection of the library was done using a ratio of beads to the PCR product of 0.6×. Briefly, after library amplification, 45 µl library was mixed with a 27 µl solution of SPRIselect beads (0.6×), then incubated at room temperature for 5 min. The microtube containing the library was then placed onto a magnetic stand to pellet the beads. The supernatant was discarded, and the beads were washed twice with 200 µl freshly prepared 80 % ethanol. The beads were left to air-dry, then resuspended in 30 µl nuclease-free water. The microtube was removed from the magnetic stand, and incubated at room temperature for another 5 min. The microtube was then placed back onto the magnetic stand to pellet the beads. The purified library was transferred into a new microtube, and brought through the 0.6× size-selection step one more time. We refer to these libraries as: ‘Ec.HF_06x.B3’; ‘Pa.HF_06x.B3’; ‘Sa.HF_06x.B3’. All libraries described here were sequenced on a MiSeq v3 600 cycle cartridge, generating 2×300 bp reads.

### Barcode design

With the purpose of upscaling the number of combinations for Illumina library preparation from 384 libraries to 9216, we designed barcodes. We generated two designs of barcodes, which we refer to as barcodes v0 and barcodes v1 (Table S2). Both designs consist of i5 (*n*=96) and i7 (*n*=96) oligo sequences, where each sequence measures 8 bp in length. In the barcode v0 design, the i5 and i7 oligos are the reverse complement sequences of each other, whereas the barcode v1 design does not include complement sequences. As it is not recommended to use the tandem complement barcodes on the NovaSeq platform (Illumina technical support, personal communication), only 9120 of the 9216 possible barcode combinations can be used out of the barcode v0 design barcodes. Hairpin, homodimer and heterodimer formation of the entire barcode sequences (F5 +i5 oligo +N5 or F7 +i7 oligo +N7) for both barcode designs (v0 and v1) were examined using the OligoAnalyzer tool from the Integrated DNA Technologies (IDT) website interface (https://sg.idtdna.com/pages/tools/oligoanalyzer) (Table S2).

The performance of both barcode designs (v0 and v1) was tested by constructing two sets of 96 Hackflex libraries, where all libraries were generated from *E. coli* genomic DNA, and each library was constructed using a distinct set of barcodes. For both designs, barcode sequences were designed such that no barcode contained three or more identical bases in a row. The barcode v0 set of libraries was sequenced on a MiSeq v2 300 cycles cartridge, 2×150 bp. The barcode v1 set of libraries was sequenced on a MiSeq v3 600 cycles cartridge, 2×300 bp.

### Hackflex v1 barcode oligo design

Hackflex v1 barcode oligos were designed to satisfy a particular set of design criteria, namely:

- Distinct from currently used length eight barcodes from common library kits (Illumina TruSeq, NEB);
- Do not contain homopolymers and certain sequences thought to be associated with sequencing error on some versions of Illumina chemistry [7] (AAA, CCC, GGG, TTT, ACA, CAC, GTG, TGT, GGCAG, GGCCG, GGCGG, GGCTG);
- Have a minimum edit distance of three from each other;
- Do not result in an oligo that forms a hairpin with a *T*_m_ >51 °C;
- Do not result in an oligo that forms a homodimer with a *T*_m_ >58 °C;
- Do not produce heterodimers between i5 and i7 that exceed a particular threshold *T*_m_.

A custom python script was implemented, using the Primer3 python API for thermodynamic calculations [8], which applied these design criteria to select a final set of 96× i5 and 96× i7 Hackflex v1 barcode oligos. The script is available at https://github.com/GaioTransposon/Hackflex.

### Nanopore library preparation and sequencing

The genomic reference of *E. coli* MG1655, against which libraries were mapped, was generated using both Illumina and Oxford Nanopore sequencing data. For long-read MinION sequencing, libraries were prepared using the 1D ligation sequencing kit (SQK-LSK108) from Oxford Nanopore Technologies (ONT) with modifications to the standard ONT protocol as described previously [6]. Samples were barcoded using the native barcoding expansion kit (EXP-NBD103) and barcoded templates were then pooled together with two other samples from an unrelated project. The final library was loaded onto a ONT MinION instrument with a FLO-MIN106 (R9.4) flow cell and run for 48 h as per the manufacturer’s instructions.

### Data availability

**Fig. 2.**
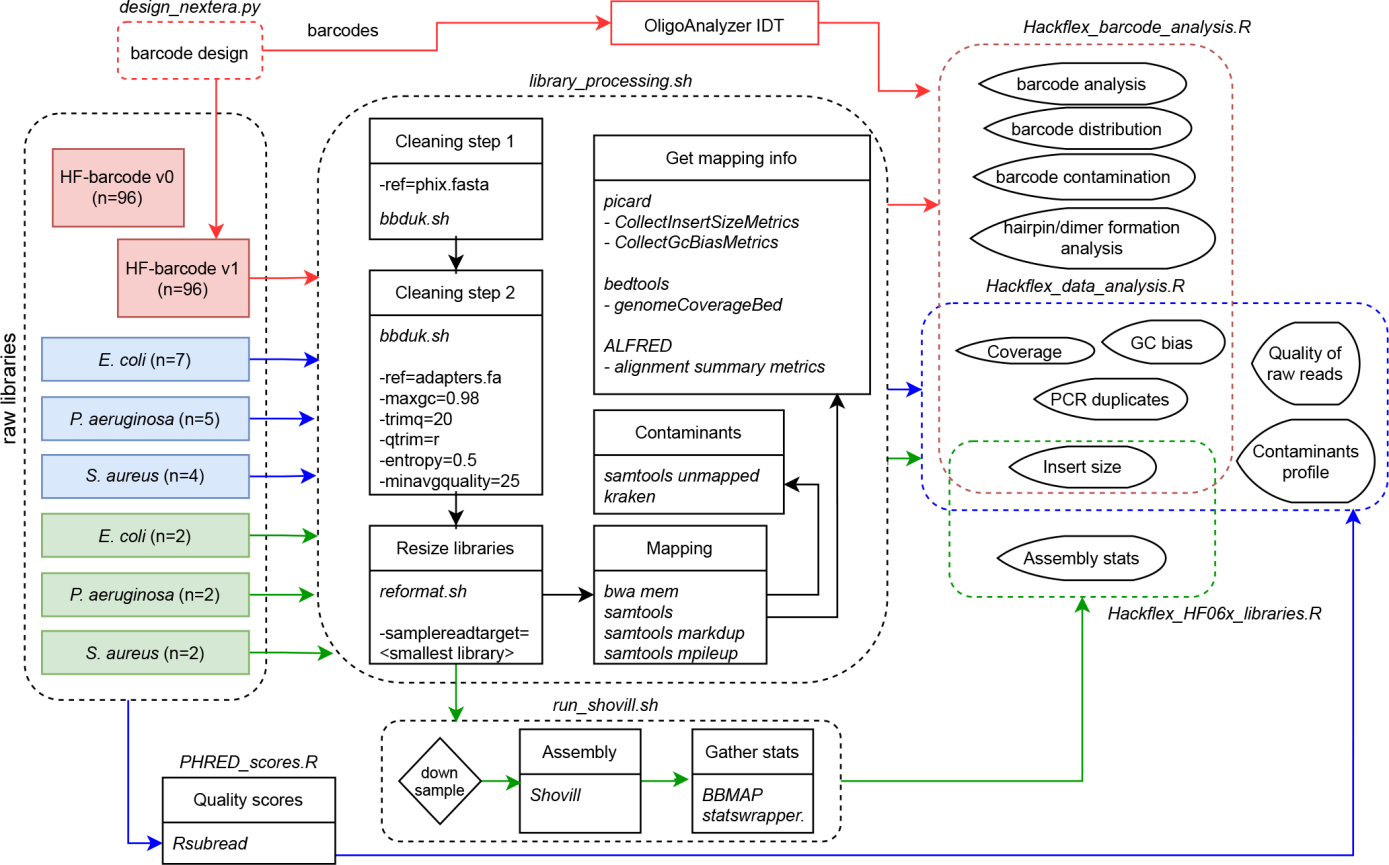
Workflow. Schematic overview of library processing and data analysis performed in this study.

All sequence data has been deposited with the National Center for Biotechnology Information under BioProject accession number PRJNA549801. The workflow including the library processing steps and the data analysis are schematically illustrated in Fig. 2.

### Barcode demultiplexing

Reads were demultiplexed with bcl2fastq (bcl2fastq 2.18.0.12; Illumina) software with default settings, allowing one mismatch per index. Barcode counts were retrieved from the demultiplexing statistics output of bcl2fastq and histograms representing barcode distribution were generated with RStudio, version 1.1.463 (RStudio: Integrated Development Environment for R). Barcode cross-contamination was assessed for barcodes v1 libraries. Barcode cross-contamination was determined by demultiplexing the data, using all possible barcode v1 combinations (*n*=9216). The rate of barcode cross-contamination is determined as the number of reads attributed to unexpected combinations (i5–i7 combos not used for the run) over the total number of reads assigned to both expected and unexpected combinations. In this manner, it is possible to distinguish the proportion of reads that failed to demultiplex because of sequencing error from those that contributed to barcode cross-contamination.

### Processing of libraries before mapping

Raw reads were assessed for quality with FastQC version 0.11.8 (http://www.bioinformatics.babraham.ac.uk/projects/fastqc/) and using the Bioconductor package Rsubread [6]. Libraries were processed using the BBTools package (http://jgi.doe.gov/data-and-tools/bbtools) (version 38.22). PhiX removal was performed (parameters: k=31 hdist=1), and adapter removal, quality trimming and filtering were performed on all the libraries with BBDuk (parameters: ktrim=r k=21 mink=11 hdist=1 tpe tbo maxgc=0.98 qtrim=rl qtrim=20 entropy=0.5). Libraries were down sampled with the bbmap script *reformat.sh* to match the total number of bases of the smallest library included in the comparison group (parameter -samplebasestarget).

### Generation of reference assemblies

Reference assemblies for library quality control were generated either via *de novo* assembly of short reads or via hybrid assembly with Nanopore sequence data, where available. For *E. coli *, Nanopore data was co-assembled with Nextera Flex libraries Ec.SF.B1 and Ec.SF_1 : 50.B1 with Unicycler software (version 0.46) with default parameters in ‘normal’ mode. For *P. aeruginosa *, the Nextera Flex libraries Pa.SF.B1 and Pa.SF_1 : 50.B1 were used. The final reference genomes obtained from the assemblies consisted of 5 (L50=1; N50=4 640 167), and 47 (L50=6; N50=350 586) contigs for *E. coli* and *P. aeruginosa *, respectively. For *S. aureus *, the reference genome available at BioSample accession number SAMN03009478 was used, which consisted of two contigs (L50=1; N50=2 778 854).

### Assembly of longer insert size Hackflex libraries

The three double-left clean-up Hackflex libraries (Ec.HF_06x.B3, Pa.HF_06x.B3, Sa.HF_06x.B3), together with the Hackflex libraries (Ec.HF.B3, Pa.HF.B2, Sa.HF.B2), were resized with *reformat.sh* (as described above), so as to obtain an equal number of base pairs for each library, and they were separately *de novo* assembled using shovill [9] (version 1.1.0). Assembly was performed with libraries down sampled at several depths (parameter --depth): 5×, 10×, 20×, 50×, 100×, 150× and 200×. Stats were run on the produced assemblies with *statswrapper.sh* (http://jgi.doe.gov/data-and-tools/bbtools) (version 38.22).

### Short-read mapping and coverage analysis

In order to assess performance of the library prep methods, libraries were aligned with bwa mem [10] (http://jgi.doe.gov/data-and-tools/bbtools) (version 38.22) alignment software and SAMtools [11] (version 1.9) (http://samtools.sourceforge.net/) to the reference genomes. After mapping, PCR duplicates were removed using the SAMtools command markdup. SAMtools mpileup was used to generate the mapped depth of coverage information for each position on the reference genomes (Fig. 2).

Insertion fragment data was obtained with picard.jar *CollectInsertSizeMetrics* (version 2.25.0-5) (http://broadinstitute.github.io/picard/) (parameters: M=0.4). Coverage by G+C content was obtained with picard.jar *CollectGcBiasMetrics* (version 2.25.0-5) (http://broadinstitute.github.io/picard/) (default parameters). Coverage across the genome was obtained with picard.jar *CollectWgsMetrics* (version 2.25.0-5) (http://broadinstitute.github.io/picard/) (parameters: READ_LENGTH=300). Bedgraphs to report coverage across all sites of the reference genomes were obtained with genomeCoverageBed (bedtools v2.27.1) (parameters: -d -ibam). Mapping files were processed with alfred [12] from which the alignment summary metrics were extracted. Reads that failed mapping were extracted with SAMtools view (version 1.9) (http://samtools.sourceforge.net/) (parameters:-f 0 x4) and analysed with Kraken2 [13] (version 2.0.8-beta) against the minikraken2_v1_8 GB database (default parameters) (Fig. 2). The contaminants profiles are reported in Table S2.

All data was processed and visualized with RStudio, version 1.1.463 (RStudio: Integrated Development Environment for R). All scripts used for the analysis and generation of plots are available at https://github.com/GaioTransposon/Hackflex.

## Results

### Quality of raw reads

Quality scores from all libraries were analysed using the Bioconductor package Rsubread [14]. For each species, mean phred scores were lower and standard deviations were higher for batch 1 libraries compared to batch 2 libraries. However, the only true direct comparison is from *E. coli* genomic DNA where standard Flex and 1 : 50 Flex libraries were made in two batches (batch 1 and batch 2). Batch 1 libraries showed a lower phred score and higher standard deviation than batch 2 libraries. phred scores were similar between standard Flex (batch 2) and Hackflex (batch 3) libraries, for libraries made from *E. coli* genomic DNA (Tables 2 and S2).

**Table 2.** Overview of raw libraries, quality scores, and number of reads obtained and filtered, with standard Flex, diluted Flex and Hackflex

Library name	phred score (mean)	phred score (sd)	Read count	Removed reads (%)	Size after resizing (bp)
Ec.SF.B1	30.47	6.01	1 000 824	3.65	174 760 709
Ec.SF_1 : 50.B1	29.9	6.85	1 200 710	3.06	175 814 069
Ec.SF.B2	33.24	3.86	1 837 948	5.42	175 540 961
Ec.SF_1 : 50.B2	32.33	4.68	1 955 102	4.8	175 569 760
Ec.HF.B3	33.1	5.38	1 486 078	3.31	175 506 039
Pa.SF.B1	28.95	6.93	974 714	4.64	148 086 604
Pa.SF_1 : 50.B1	28.29	7.4	1 581 282	5.26	149 084 666
Pa.HF.B2	31.87	4.89	1 631 262	4.67	148 779 715
Sa.SF.B1	32.87	4.53	779 660	1.74	166 527 991
Sa.SF_1 : 50.B1	32.17	5.15	1 090 564	1.97	167 353 079
Sa.HF.B2	33.95	3.46	4 236 794	3.14	167 220 961

### How adapter trimming and quality filtering affected distinct libraries

The cleaning step, consisting of adapter trimming and quality filtering, affected libraries made from *E. coli* genomic DNA with either standard Flex or the Hackflex protocol in a similar manner. However, a higher percentage of reads was removed from batch 2 than from batch 1 libraries (Ec.SF.B1, 3.65 %; Ec.SF_1 : 50.B1, 3.06 %; Ec.SF.B2, 5.42 %; Ec.SF_1 : 50.B2, 4.80 %). Libraries from *P. aeruginosa* genomic DNA had 4.64, 5.26 and 4.67 % of the reads removed during the cleaning step, for standard Flex, 1 : 50 diluted Flex and Hackflex, respectively. Libraries from *S. aureus* genomic DNA had a higher percentage of reads removed from the Hackflex (3.14 %) compared to standard Flex (1.74 %) and 1 : 50 diluted Flex (1.97 %) library (Table 2). In order to compare the performance of the library protocols, within each species all libraries were resized based on the smallest library, so as to obtain an equal number of base pairs for each library (Table 2). We report the mean read length, and the number of reads and base pairs obtained from each library, before and after the cleaning steps, in Table S2.

Given the relatively small amount of sequencing data required for microbial isolate genome sequencing, PCR duplicates would be expected to be very rare in standard Nextera Flex reactions, which can prepare human genomes for shotgun sequencing (30× coverage of a 3 Gbp genome). However, by reducing the amount of BLT and increasing the number of PCR cycles used in Hackflex, the library complexity is expected to be reduced and, therefore, a higher incidence of PCR duplicates may occur. To understand the impact of Hackflex on PCR duplicates, we compared the PCR duplicates obtained with standard Flex, 1 : 50 diluted Flex and Hackflex. We do not observe an excess of PCR duplicates in 1 : 50 diluted and Hackflex libraries compared to standard Flex libraries, at least at the sequencing depth tested (Tables 3 and S2).

**Table 3.** Mapping details for libraries generated with standard Flex, diluted Flex and Hackflex

Library name	Unmapped reads	Mapped fraction	Mismatch rate	PCR duplicates	Coverage (mean)	Coverage (sd)
Ec.SF.B1	170	0.989	0.002	10 906	36.22	13.41
Ec.SF_1 : 50.B1	164	0.992	0.002	8655	36.55	12.77
Ec.SF.B2	53	0.991	0.001	6998	36.4	13.08
Ec.SF_1 : 50.B2	134	0.993	0.001	5288	36.76	11.14
Ec.HF.B3	114	0.994	0.001	4738	36.46	12.49
Pa.SF.B1	107	0.994	0.002	5678	23.4	7.14
Pa.SF_1 : 50.B1	160	0.995	0.002	4335	23.55	7.22
Pa.HF.B2	73	0.996	0.001	2918	23.65	7.84
Sa.SF.B1	48	0.988	0.002	9255	58.56	14.32
Sa.SF_1 : 50.B1	123	0.991	0.001	7674	58.93	14.18
Sa.HF.B2	237	0.993	0.001	4627	59.03	15.14

### Coverage

Coverage profiles were obtained by mapping the equally resized libraries against their reference genomes. As expected, a highly similar median coverage was obtained among libraries made using the different protocols, independently of the genomic DNA source. Mapped read fractions, mean coverage depth and other mapping metrics for *E. coli *, *P. aeruginosa* and *S. aureus* libraries were similar among standard Flex, diluted Flex and Hackflex libraries (Tables 3 and S2).

### Low coverage regions

Standard Flex (batches 1 and 2), 1 : 50 Flex (batches 1 and 2) and Hackflex libraries from *E. coli* genomic DNA had no sites with zero coverage. Libraries made from *S. aureus* genomic DNA had 0, 0 and 1 sites with zero coverage (near an extremity of one contig), for standard Flex, 1 : 50 Flex and Hackflex libraries, respectively. Libraries made from *P. aeruginosa* genomic DNA had considerably more sites with zero coverage than libraries from *E. coli* and *S. aureus *, possibly due to the fragmented reference genome. In fact, there were 651, 263 and 13 341 sites with zero coverage, for standard Flex, 1 : 50 Flex and Hackflex libraries, respectively. The low coverage regions were overlapping to a certain extent among the three libraries, possibly indicating common features of these regions that bias against their sequencing with Illumina chemistry. Genomic sites with zero coverage for all libraries are reported in Table S2.

### Barcode distribution and quality

Two sets of 96 i5 and i7 oligos (8 bp), referred to as barcodes v0 and v1, were designed for this study to provide a resource for high-throughput multiplexing of Hackflex libraries. To this end, performance of the two sets of 96 designed barcodes was evaluated by subjecting *E. coli* MG1655 DNA to 2×96 independent library constructions with Hackflex reagents, each library with a different barcode combination. Barcode distribution for both barcode designs and G+C bias is displayed in Fig. S1.

### Barcodes v0

The 96 libraries with the barcode v0 design are made using the tandem complement design, and we refer to these libraries as Ec.HF-barcode_v0(t).B0, where ‘v0’ is to indicate the v0 barcode design and the ‘(t)’ is to indicate that these libraries were constructed using i5 and i7 oligos that are tandem complements of each other. Of these 96 libraries, 2 had failed (read count *n*=12 and *n*=18). The mean barcode count was 11 033.8 (sd=4149.3). The coefficient of variation was 0.38 and 0.34 including all libraries and excluding the two failed libraries, respectively. The mean G+C content of oligos is 49.87 (median=50) and 2 of the 192 v0 oligos have a G+C content of 87.5 %. The G+C content of the entire barcode sequence (i.e. F5 +i5+N5 or F7 +i7+N7) was plotted against the read count obtained from each barcode, to estimate the extent of G+C bias. A significant correlation between read counts obtained and G+C content was found for i5 (*R*=−0.704; *P*<0.0001) and i7 (*R*=−0.704; *P*<0.0001) (Fig. S1).

Barcodes v0 had an estimated ΔG between −10.7 and −4.2 kcal mol^−1^ (−44.8 and −17.6 kJ mol^−1^), and their mean melting temperature was 50.36 °C (sd=4.56), which is over 10 °C lower than the annealing temperature used in the PCR reaction (62 °C) (Table 1). We examined the hairpin formation, and homodimer and heterodimer formation of the entire barcodes used to build the two failed libraries. These libraries had a normal estimated ΔG (mean=−6.45 kcal mol^−1^, sd=0.73) and a melting temperature that approached the 62 °C annealing temperature used in the Hackflex protocol (C3_i5=55.5 °C; C3_i7=48.6 °C; H7_i5=51.2 °C; H7_i7=58.1 °C). These two libraries were analysed for hairpin, homodimer and heterodimer formation. A hairpin formation was found for an i5 oligo (C3_i5 ΔG=−5.5 kcal mol^−1^ ; *T*_m_=55.5 °C) and for one i7 oligo (H7_i7 ΔG=−7.02 kcal mol^−1^ ; *T*_m_=58.1 °C), possibly explaining the failure of these two libraries (Table S2). However, the same two barcode combinations were used in another project (BioProject accession number PRJNA526405) where they produced successful libraries (BioSample accession numbers SAMN11098202 and SAMN11098246), suggesting that the failures seen here may not be due to a faulty barcode design. Further data would be required to resolve whether the barcode oligos in question produce libraries with lower efficiency.

### Barcodes v1

**Fig. 3.**
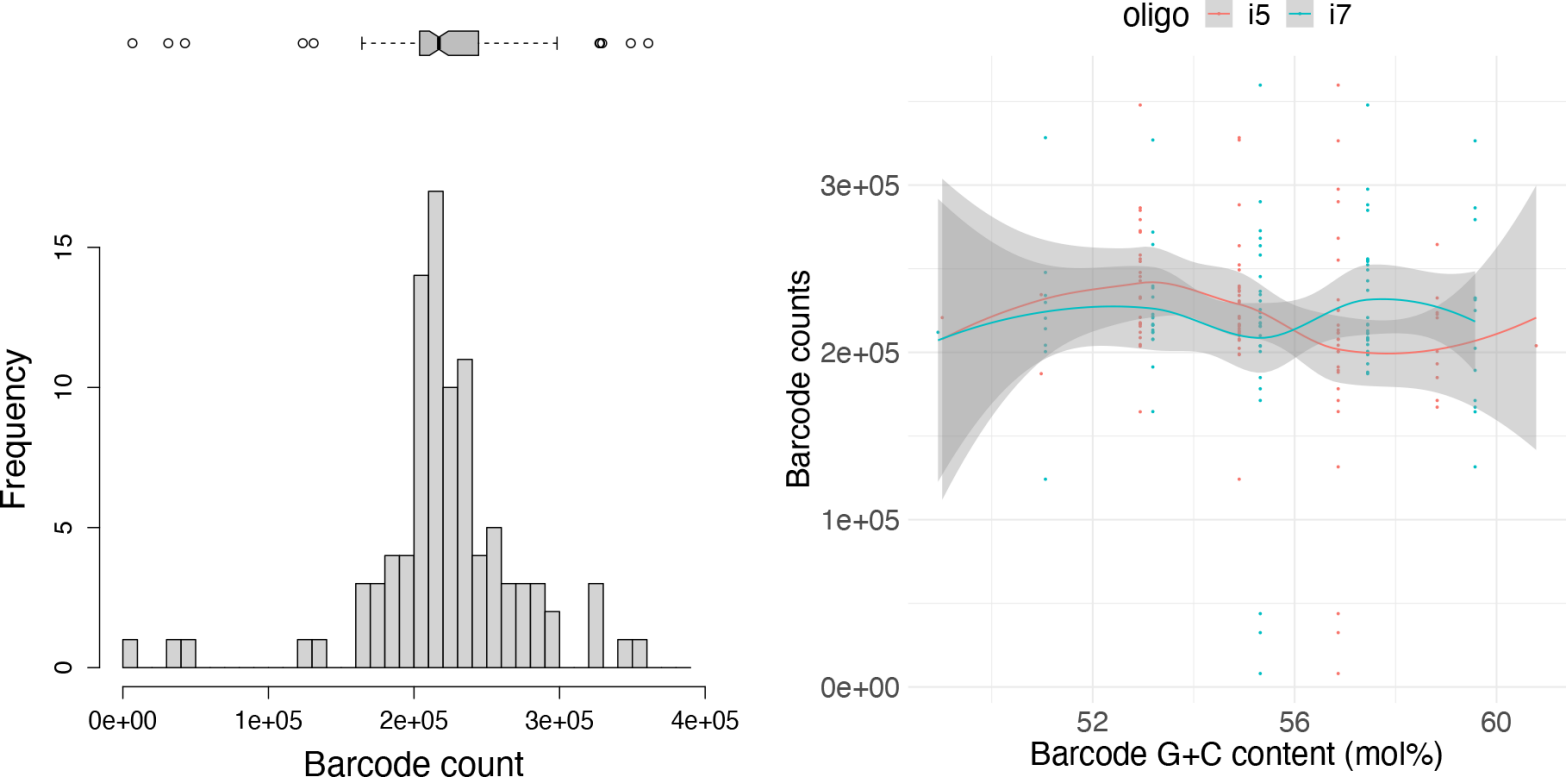
Barcode distribution and G+C bias of Hackflex barcode v1 libraries. Unique barcode distribution across 96 Hackflex libraries constructed from barcodes v1 (left) and their G+C bias: the relation between barcode counts obtained for each entire barcode (i.e. F5 +i5+N5 or F7 +i7+N7), and its G+C content (i5, *R*=−0.17, *P*=0.1; i7, *R*=0.03, *P*=0.77) (right).

The 96 libraries made from barcodes v1 do not follow the tandem complement design. The mean barcode count was 221 203.1 (sd=53 836.6). The coefficient of variation was 0.243 and 0.183 including all libraries and excluding the three libraries with low counts (8036; 32 469; 43 836), respectively. The mean G+C content of oligos is 39.65 (median=50) and two v1 oligos have a G+C content of 0. The G+C content of the entire barcode sequence (i.e. F5 +i5+N5 or F7 +i7+N7) was plotted against the read count obtained from each barcode, to estimate the extent of G+C bias. No correlation was found between read counts obtained and G+C content of either i5 (*R*=−0.17; *P*=0.1) or i7 barcode (*R*=0.03; *P*=0.77) (Fig. 3). The observed barcode cross-contamination rate was 0.002. Assuming uniform cross-contamination, this would result in a sample misassignment rate ranging between 0 and 0.2 % of reads, depending on which and how many barcodes are used in a pool of samples.

The estimated ΔG ranged between −7.660 and −4.220 kcal mol^−1^ (−32.0 and −17.7 J mol^−1^), and the mean melting temperature was 49.07 °C (sd=3.32), which is over 10 °C lower than the annealing temperature used in the PCR reaction (62 °C) (Table 1). We examined the hairpin formation, homodimer and heterodimer formation of the entire barcodes used to build the three libraries that yielded a low read count. These libraries had similar ΔG (mean=−5.72 kcal mol^−1^, sd=1.2) and similar melting temperatures (mean=49.6 °C, sd=1.24) to the rest of the libraries. However, one i5 oligo (v1_A1_i5) was found to form hairpins (ΔG=−7.06 kcal mol^−1^ ; *T*_m_=51.6 °C) and one i7 oligo (v1_G2_i7) was found to form a homodimer structure consisting of homodimerization with itself (5 bp) and homodimerization between F7 and N7 (5 bp) in its proximity (ΔG=−13.41 kcal mol^−1^ ; *T*_m_=48.6 °C) (Table S2).

### Insert size

Insert size distribution was analysed for all libraries. Small differences in insert size distribution were detected between different batches, and although standard Flex libraries from *P. aeruginosa* and *S. aureus* had larger insert sizes compared to diluted 1 : 50 libraries, an equally large or larger insert size was found in Hackflex libraries compared to standard Flex libraries, in all species (Table 4).

**Table 4.** G+C content coverage bias and insert size lengths generated with standard Flex, diluted Flex and Hackflex

Library name	Insert size (median) (bp)	Insert size (mean) (bp)	G+C bias (Pearson’s *R*)	*P* value
Ec.SF.B1	302	296.31	−0.886	***
Ec.SF_1 : 50.B1	230	238.68	−0.8	***
Ec.SF.B2	326	334.12	−0.383	**
Ec.SF_1 : 50.B2	291	296.16	−0.598	***
Ec.HF.B3	287	300.77	−0.763	***
Pa.SF.B1	269	266.97	−0.965	***
Pa.SF_1 : 50.B1	241	242.39	−0.965	***
Pa.HF.B2	341	351.72	−0.248	*
Sa.SF.B1	293	293.1	0.698	***
Sa.SF_1 : 50.B1	269	270.79	0.099	NS
Sa.HF.B2	296	304.74	0.741	***

* p < 0.05

** p < 0.01

*** p < 0.001

NS, Not significant.

The additional 96 HF barcode v1 libraries that were generated from *E. coli* DNA to assess the performance of barcodes underwent a pooled bead clean-up, and were analysed to assess the insert size distributions obtained with the Hackflex protocol. With the exclusion of the 3 libraries with low counts, 93 libraries had a mean insert size of 310.60 bp (range of library means, 297.66–314.57; sd range, 120.66–133.29) (Table S2).

### G+C content bias

In order to assess the extent of G+C content bias, correlations were obtained between coverage and G+C content across the genome using the weighted Pearson’s correlation coefficient (*R*) on the relative (observed/expected) measures of sequence coverage by the reads at each G+C content window. All libraries showed, to a certain extent, a bias at extreme G+C content areas as it would be expected [15, 16]. The dilution factor and, hence, number of PCR cycles did not have an obvious association with strength of G+C bias, but the G+C bias seemed to be associated with batch effects (Ec.SF.B1 *R*=−0.886; Ec.SF_1 : 50.B1 *R*=−0.8; Ec.SF.B2 *R*=−0.383; Ec.SF_1 : 50.B2 *R*=−0.598; Pa.SF.B1 *R*=−0.965; Pa.SF_1 : 50.B1 *R*=−0.965). In libraries from *S. aureus* genomic DNA, the G+C bias was similar between standard Flex and Hackflex (Sa.SF.B1 *R*=0.698; Sa.HF.B2 *R*=0.741; test statistic *z*=−0.509, *P*=0.305), and in libraries from *P. aeruginosa* genomic DNA, the G+C bias was lower for Hackflex than for standard Flex or 1 : 50 Flex (Pa.SF.B1 *R*=−0.965; Pa.SF_1 : 50.B2 *R*=−0.965; Pa.HF.B2 *R*=−0.248; test statistic *z*=−10.037, *P*<0.0001) (Table 4)

Again, as for the insert size distribution range of Hackflex, we used the Hackflex barcode v1 libraries from *E. coli* genomic DNA to estimate the G+C bias with the Hackflex protocol on a large number of libraries. As a high coverage depth can offer a better estimate of G+C bias, we included the top 20 Hackflex barcode v1 libraries with the highest read count in the analysis (mean depth after resizing of libraries: 12.5×). The mean G+C bias (Pearson’s *R*) found was −0.642 (sd=0.043; *var*=0.00188; median=−0.650).

### Size selection on Hackflex libraries can improve *de novo* assembly

**Fig. 4.**
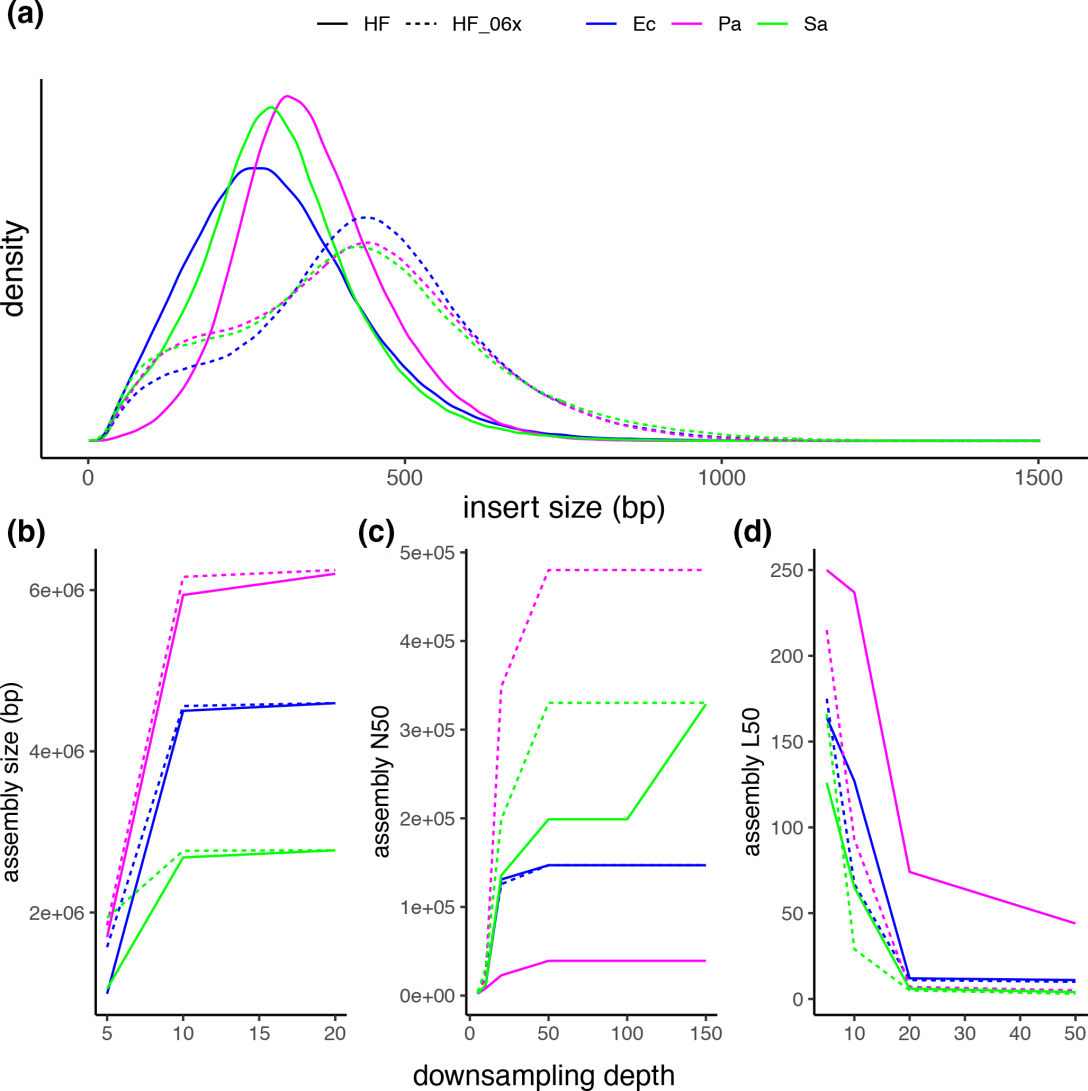
Insert size distribution and assembly metrics of double-left clean-up Hackflex libraries compared to Hackflex libraries. Insert size distributions (a) are shown for Hackflex and double-left clean-up Hackflex libraries from *E. coli* (blue) (Ec.HF.B3 median=287, mean=300.68; Ec.HF_06x.B3 median=433, mean=432.72), *P. aeruginosa* (pink) (Pa.HF.B2 median=341, mean=352.01; Pa.HF_06x.B3 median=417, mean=415.06) and *S. aureus* (green) (Sa.HF.B2 median=296, mean=304.94; Sa.HF_06x.B3 median=416, mean=420.29) genomic DNA. The same libraries, down sampled to several depths, and assembled, were analysed for assembly quality. Assembly size (b), and assembly metrics N50 (c) and L50 (d) are shown for Hackflex and double-left clean-up Hackflex libraries from *E. coli* (blue), *P. aeruginosa* (pink) and *S. aureus* (green).

We generated three Hackflex libraries by performing a double-left bead clean-up. For standard Hackflex libraries, a median insert size of 287–341 bp was obtained, whereas Hackflex libraries that included a double-left clean-up step resulted in an increased median insert size of 416–433 bp (Fig. 4).

To evaluate the effects of coverage depth as it relates to the influence of insert size, assemblies were performed from libraries down sampled to various coverage depths. Assemblies from Hackflex libraries were more fragmented (higher L50) compared to double-left clean-up Hackflex libraries. The effect was most prominent in libraries from *P. aeruginosa *. Resizing of the two libraries brings the depth to 33 and 34 mean coverage for Pa.HF_06x.B3 and Pa.HF.B2, respectively. Down sampling the two libraries to the common maximum depth achieved (33×), shows that a significant difference in assembly quality between protocols was detectable (Pa.HF.B2 N50=39210, L50=44; Pa.HF_06x.B3 N50=480098, L50=5). Similarly, the difference between the two protocols was prominent in libraries from *S. aureus *, where a larger N50 was obtained in the double-left clean-up Hackflex library compared to the Hackflex library at either 10× (Sa.HF.B2 N50=11756, L50=65; Sa.HF_06x.B3 N50=30473, L50=29), 20× (Sa.HF.B2 N50=135652, L50=6; Sa.HF_06x.B3 N50=198717, L50=5), or 50× (Sa.HF.B2 N50=198879, L50=4; Sa.HF_06x.B3 N50=330244, L50=3). Down sampling the two libraries to the common maximum depth achieved (median=116) shows that a similar assembly quality is reached (Sa.HF.B2 N50=328703, L50=3; Sa.HF_06x.B3 N50=330244, L50=3). In *E. coli* libraries, the benefit of larger insert sizes libraries on assembly quality was prominent at 10× depth (Ec.HF.B3 N50=10798, L50=127; Ec.HF_06x.B3 N50=21488, L50=67), mild at 20× depth (Ec.HF.B3 N50=131011, L50=12; Ec.HF_06x.B3 N50=125707, L50=11), and non-existent at 48× depth (Ec.HF.B3 N50=147079, L50=11; Ec.HF_06x.B3 N50=147079, L50=10) (Fig. 4).

Other metrics were not affected by the double-left clean-up. Equally resized libraries Ec.HF.B3 and Ec.HF_06x.B3 had a mean coverage of 46.9 and 46.8, respectively, with no sites with zero coverage. These libraries had a 0.66 correlation in coverage (*P*<0.0001), similarly to Ec.HF.B3 and Ec.SF_1 : 50.B2 (Pearson’s *R*=0.58; *P*<0.0001). Equally resized libraries Pa.HF.B2 and Pa.HF_06x.B3 had a mean coverage of 33.55 and 33.24, with 7755 and 195 sites with zero coverage, respectively. These libraries had a 0.20 correlation (*P*<0.0001), similarly to Pa.HF.B2 and Pa.SF_1 : 50.B2 (Pearson’s *R*=0.14; *P*<0.0001). Equally resized libraries Sa.HF.B2 and Sa.HF_06x.B3 had a mean coverage of 117.03 and 116.09, respectively, with no sites with zero coverage. These libraries had a 0.42 correlation (*P*<0.0001), similarly to Sa.HF.B2 and Sa.SF_1 : 50.B2 (Pearson’s *R*=0.44; *P*<0.0001).

### Additional libraries

Additional libraries were prepared in order to test the effect of PrimeSTAR on the standard Flex protocol, and the use of different annealing and extension temperatures in the Hackflex protocol. A lower annealing temperature was associated with a stronger G+C bias in both *P. aeruginosa* (Pa.HF_55A.B2 *R*=−0.734, *P*<0.0001; Pa.HF.B2 *R*=−0.248, *P*=0.048) and in *S. aureus* libraries (Sa.HF_55A.B2 *R*=0.945, *P*<0.0001; Sa.HF.B2 *R*=0.741, *P*<0.0001). A lower annealing temperature and a higher extension temperature in the Hackflex protocol, only tested with *P. aeruginosa* genomic DNA, was associated with an even higher G+C bias (Pa.HF_55A72E.B2 *R*=−0.851, *P*<0.0001; Pa.HF.B2 *R*=−0.248, *P*=0.048) (Table S2).

## Discussion

Our customized library preparation protocol, Hackflex, involves several modifications to the standard Nextera Flex method (recently renamed as Illumina DNA Prep), including the use of a 1 : 50 dilution of the BLT and the replacement of several kit components with alternative reagents to greatly expand the total number of libraries that can be produced from a single kit. Previous cost-reducing methods were based on the reduction of volumes [2, 3, 5]. However, working with small volumes can be problematic because of evaporation, whether the process is carried out manually or using robotics. When using liquid-handling robots, this issue can be solved by creating dead volumes, but the dead volume for many low-cost liquid handling robots is high enough (usually more than 10 % of what needs to be pipetted) that it may waste significant amounts of expensive undiluted reagents, thereby naturally increasing the costs. The process of making the Hackflex reagents can take up to 3 h. However, Hackflex reagents can be made in batches, with a batch used over a period of 1 year. To evaluate the performance of the Hackflex protocol, we constructed libraries in parallel using the standard Nextera Flex protocol (referred to as standard Flex), an adapted version of the Nextera Flex protocol using the diluted transposase beads (referred to as 1 : 50 Flex) and using the Hackflex protocol. Libraries were constructed from the genomic DNA of either *E. coli* MG1655, *P. aeruginosa* PAO1 or *S. aureus* ATCC 25923.

### Barcode designs

To enable greater flexibility and higher multiplexing in sample barcoding, we designed our own barcode sequences, produced by Integrated DNA Technologies (IDT) using their standard oligo plate manufacturing process. We generated two designs: v0 and v1. In the barcode v0 design, i5 oligos are the tandem complement sequences of the i7 oligos. Although the tandem design of v0 barcodes did not significantly impact the quality of the data on a MiSeq instrument, a significant degradation of index read two quality (i5) was observed when such barcode combinations were applied to generate data for a large-scale, porcine-gut, metagenomics project [17, 18]. It was noted by Illumina technical support that the tandem complement design could lead to significantly reduced quality scores for the i5 index read on the NovaSeq instrument (Illumina technical support, personal communication). Therefore, we designed new barcodes, which we refer to as barcodes v1, that do not have a tandem complement design and we tested their performance by constructing 96 Hackflex libraries, from 96 barcode combinations.

Incorrect sample barcode assignment can occur due to barcode cross-contamination during sample preparation or sequencing, or it can be due to errors during sequencing such as the presence of multiple overlapping clusters on the flow cell surface, base miscalls and image processing errors during cluster calling. Sample barcode misassignments can be minimized if sample barcode oligos are designed to be sufficiently unique [19], where more than 3 bp must be errors for the barcode to be misallocated. The use of unique dual indexing where each of i5 and i7 index sequences are unrelated to each other is also known to mitigate sample barcode misassignment issues (https://support.illumina.com/bulletins/2018/08/understanding-unique-dual-indexes--udi--and-associated-library-p.html). From our v1 barcodes, we report a barcode cross-contamination rate of 0.002 for our new barcodes. How this translates into sample misassignment of reads will depend heavily on the number of samples in a pool and the specific barcode oligos in use. This low rate of sample misassignment should be sufficient for consensus sequencing applications such as *de novo* genome assembly, where the impact of a low rate of misassigned reads is likely to be very limited.

Three out of the ninety-six v1 barcode libraries yielded a low read count. One possible cause is pipetting error during various stages of the library prep where a multichannel pipette was used. Alternatively, failures could be attributable to barcode-specific reaction kinetics. One of these libraries with low read counts has an i7 barcode for which a particularly low ΔG is predicted for homodimer formation, but the remaining i5 and i7 barcodes appear indistinguishable from other barcodes in terms of G+C content, hairpin, homodimer and heterodimer thermodynamics.

While a significant negative correlation was found in the v0 design, no correlation was found in the v1 barcode design between the barcode G+C content and the read count obtained. There are several possible explanations for the variance in barcode representation in our design, including formation of homodimers and heterodimers, and hairpins. The v1 barcodes have been designed to be distinct from the current set of standard 8 bp Illumina barcode sequences. There is no reason a user of the Hackflex protocol would not be able to use standard Illumina barcode adapter oligos together with our protocol modifications; however, we have not tested performance under those conditions.

Worthy of note, the performance of Hackflex barcode libraries is assessed based on the read count obtained from each barcode set, which can be affected by the barcode oligo performance as well as by the laboratory operator. We believe that the observed performance can be interpreted as a lower bound on the performance of the oligos themselves. That is, in the absence of operator error, the numbers reflect the performance of the oligos. However, the true oligo performance could be better, given that some of the observed variation may have been introduced by the operator.

### Quality of sequence data

Hackflex uses a lower amount of input DNA (10 rather than 200 ng) compared to the standard Flex protocol. Coupled to the reduction of input DNA, a higher number of PCR cycles is used in Hackflex (12 instead of 5). This modification could be expected to increase the number of PCR duplicates. However, we did not detect a higher number of PCR duplicates in diluted libraries (i.e. Hackflex and 1 : 50 Flex) compared to undiluted libraries (i.e. standard Flex), suggesting that diluted libraries are either equally efficient as undiluted libraries, or that any apparently duplicate read pairs are derived from transposition bias or flow cell duplicates. It is likely that much deeper sequencing of these libraries would be required to reveal differences in library complexity. Raw libraries from distinct protocols (i.e. standard Flex, 1 : 50 Flex and Hackflex) showed comparable quality scores and behaviour through the adapter and quality trimming steps. To further compare the performance of distinct protocols, libraries were randomly subsampled to achieve equally sized libraries and undergo mapping against a reference genome.

As a consequence of the polymerase’s bias against regions with extreme G+C content, it can be expected that by increasing the PCR cycles, G+C bias will be increased. The G+C bias was lower for Hackflex libraries than for standard Flex and/or 1 : 50 Flex, in *S. aureus* and *P. aeruginosa *, respectively. For *E. coli *, we were able to provide an estimate of G+C bias from the top 20 Hackflex barcode v1 libraries with the highest coverage depth. The G+C bias estimate obtained from these libraries had a mean G+C bias of −0.642 (sd=0.043), which is lower than that of standard Flex and diluted Flex libraries from batch 1, and higher than that of standard Flex and diluted Flex libraries from batch 2. However, as the G+C bias estimates for standard Flex and 1 : 50 Flex from *E. coli* genomic DNA were derived from four libraries (two replicates), and the number of libraries for direct comparison for *P. aeruginosa* and *S. aureus* is also limited, we could not draw a statistically significant conclusion that Hackflex has a lower or equally low G+C bias compared to standard Flex.

Additionally, we explored other conditions for Hackflex, such as a lower annealing and a higher extension temperature. This resulted in a stronger G+C bias, but the limited number of datapoints means the result is inconclusive. An effect on the uniformity of coverage could have been expected when substituting PrimeSTAR for EPM in the Hackflex protocol. Although a batch effect was detected, the coverage obtained with the distinct protocols was highly similar within each batch, as indicated by the read mapping rates, the number of unmapped reads, the median and mean coverages, and the number of sites reporting zero coverage. Sites with zero coverage in *E. coli* and *S. aureus* were located near the extremities of contigs, raising the possibility that the coverage dropouts may be due to read mapping artefacts rather than lack of sequence data. However, a higher number of sites with zero coverage (double) were obtained for *P. aeruginosa* with Hackflex, as compared to the standard Flex protocol. We are unsure how to explain this observation given that PrimeSTAR GXL is specified for PCR of high G+C samples such as *P. aeruginosa *. However, the fact that the reference genome (against which the libraries were mapped) was assembled from the standard Flex and diluted Flex libraries could have biased the mapping in favour of those libraries and against Hackflex. Other coverage metrics were highly similar among the different protocols, and Hackflex showed nearly a fourfold lower G+C bias on the high G+C *P. aeruginosa* genome than either standard Flex or 1 : 50 Flex.

We analysed insert size distribution obtained with Hackflex and compared it to that obtained with standard Flex and 1 : 50 Flex. We did not find significant differences in insert size distributions between distinct protocols. However, as insert size distribution is highly dependent on manual pipetting and could also be affected by undocumented BLT chemistry changes across distinct batches, it is difficult to draw any conclusions from comparisons of insert size distributions between protocols across batches.

### Tunability to other size ranges

The size distribution of fragments generated by the standard Nextera Flex workflow appears to be strongly skewed towards small fragments. This is also seen in Hackflex and is likely driven by the current formulation of BLT. While the fragment sizes generated by tagmentation with transposase in solution are highly sensitive to the relative concentrations of DNA and transposomes, the fragment sizes generated from BLT appear much less sensitive to those parameters. This has the benefit of making the reaction more robust to variation in input DNA concentration, but comes at a cost in that there is less flexibility to optimize the yield of fragments in a particular size range. In particular, when carrying out *de novo* assembly, longer reads from larger inserts are known to have a beneficial effect [20]. Moreover, longer reads such as the PE250 mode offered on MiSeq and NovaSeq instruments may offer less benefit on inserts less than 500 nt in size, because PE250 on shorter inserts would generate overlapping reads on the same fragment, yielding redundant sequence data. Therefore, we evaluated the ability of Hackflex to produce larger insert libraries of sufficient complexity for *de novo* microbial genome assembly.

Hackflex libraries were made from *E. coli *, *P. aeruginosa* and *S. aureus *, where two rounds of bead clean-up were carried out to remove the small insert size. This is done by cleaning up the libraries through SPRIselect beads size selection at 0.6× ratio twice (double-left clean-up). In doing so, the fragments with small inserts were removed and the mean insert size length of the library has increased. The increase of insert size can be of benefit for assembly and, particularly, for assembly of certain species. As we ran assemblies at different depths, we compared the assemblies obtained, and concluded that the double-left clean-up step in Hackflex helps improve the assembly, in particular at lower coverage and for *P. aeruginosa *, which is a larger and more complex genome than the other two. Despite improving the assembly, the double-left clean-up did not affect the quality of the data produced, as reported by other metrics.

### Applicability to large and complex genomes

Given that the transposase dilution has a direct effect on library complexity, the Hackflex method may not be suitable for very large genomes, such as those of humans and plants. In order to increase the library complexity, the Hackflex method could be tweaked to decrease the transposase dilution. However, Hackflex is designed to enable large-scale sequencing projects for low-complexity genomes such as bacteria, including metagenomic projects with high sample numbers [17, 18] or DNA surveillance projects, where thousands of bacterial genomes must be sequenced.

### Limitations

Barcode v0 and barcode v1 design libraries were sequenced with different chemistries. Although it is not ideal to use different sequencing chemistries to test the performance of the two barcode designs, a batch effect can also be expected from different runs of the same sequencing chemistry, as the loading concentration affects the index read quality and cluster calling on the unpatterned (flat) flow-cell surface used by the MiSeq. In order to avoid these run-specific batch effects, both barcode design sets of libraries would have to be sequenced on the same run. However, in the case of barcode v0 and barcode v1 designs, where the same or a similar barcode exists in both designs, running the two sets under the same run would have caused barcode collisions and the inability to determine the rate of unassigned reads for each scheme. Furthermore, for any comparison to reach statistical power, the sample size (dictated by the number of flow cells, in this case) would become prohibitively expensive.

### Conclusion

Here, we have developed and characterized an alternative method of library construction for Illumina sequencing that, by reducing the library prep expenses, allows users to process from 9.87- to 14-fold more samples at the same reagent cost. This study demonstrates that data of comparably high quality can be obtained with Hackflex as could be generated by the existing Nextera Flex method. Comparison with the existing Nextera Flex method demonstrates that Hackflex is a valid and cost-effective alternative to construct libraries at a large scale.

## Supplementary Data

Supplementary material 1

Supplementary material 2

Supplementary material 3
