# Supplementary material for: Hackflex: low-cost, high-throughput, Illumina Nextera Flex library construction
Source: Microb Genom. 2022 Jan 11;8(1):000744. doi: 10.1099/mgen.0.000744 (PMC8914357; doi:10.1099/mgen.0.000744)
Supplement: Supplementary material 1 [file mgen-8-0744-s001.pdf]

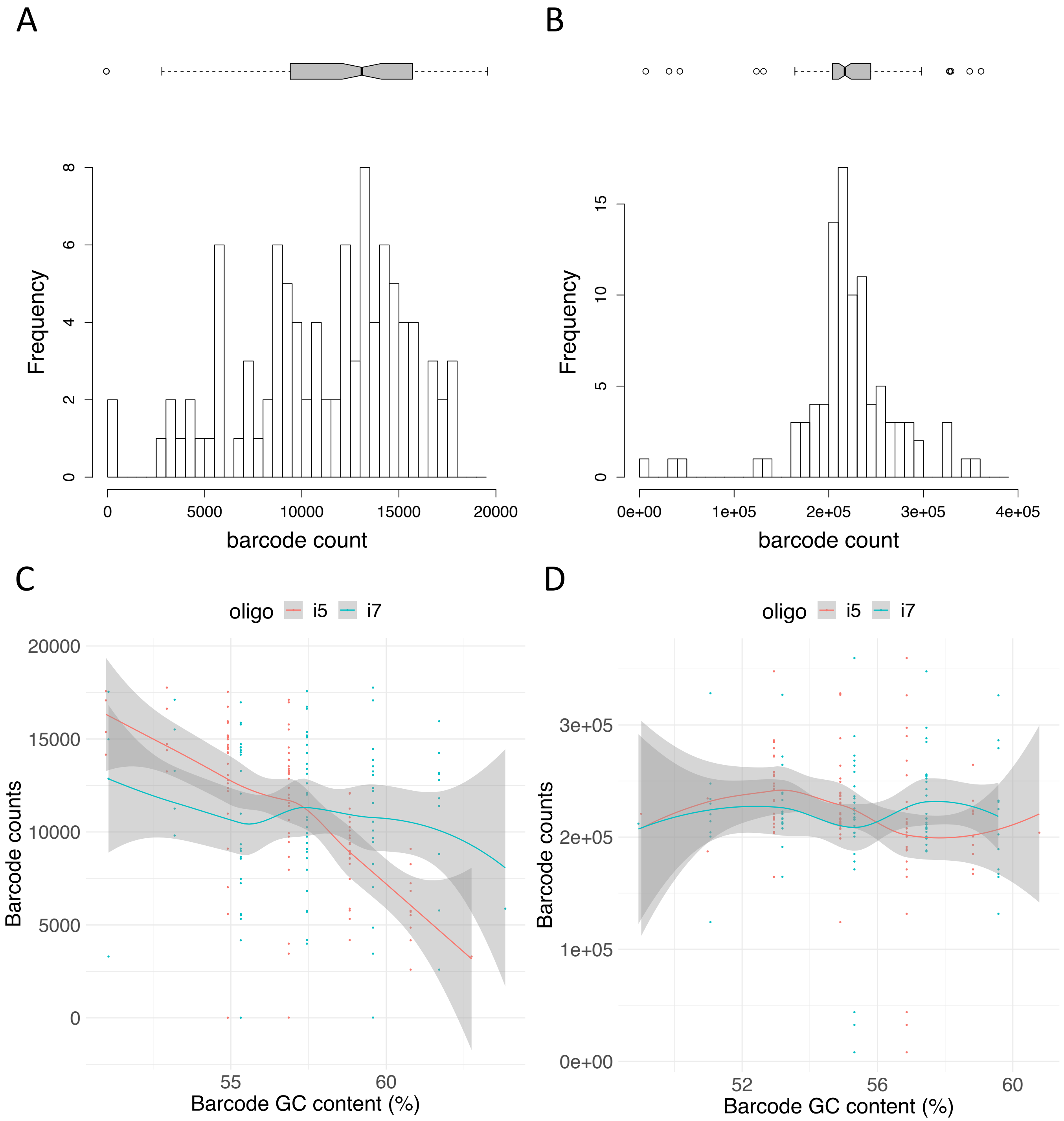

Supplementary Figure 1. Barcode distribution and GC bias.

Unique barcode distribution across 96 Hackflex libraries constructed from barcodes v0 (**A**) and 96 Hackflex libraries constructed from barcodes v1 (**B**). Bottom plots show the relation between barcode counts obtained for each entire barcode (*i.e*.: F5+i5+N5 or F7+i7+N7), and its GC content. Barcodes v0 showed a negative correlation between the barcodes GC content and the counts obtained (i5 and i7 oligo R=-0.704 p<0.0001) (**C**). Barcodes v1 showed no significant correlation between the barcodes GC content and the counts obtained (i5 R=-0.17 p=0.1; i7 R=0.03 p=0.77) (**D**).
